# Heyde's Syndrome and Transcatheter Aortic Valve Implantation

**DOI:** 10.5935/abc.20160193

**Published:** 2017-04

**Authors:** Conrado Pedroso Balbo, Luciana Paula Seabra, Victor Gualda Galoro, Guido Caputi, José Honório Palma, Ênio Buffolo

**Affiliations:** Hospital do Coração da Associação Sanatório Sírio, São Paulo, SP - Brazil

**Keywords:** Stenosis Valve Aortic / therapy, Gastrointestinal Hemorrhage / complications, Angiodysplasia, Prosthesis Implantation of Aortic Valve, von Willebrand Diseases / therapy

## Introduction

Aortic Stenosis (AoS) is the valvular pathology most frequently acquired in developed countries, present in 4% of individuals over 85 years of age. Patients with severe AoS may have comorbidities, presenting greater risk of thrombotic and bleeding events.

Heyde's Syndrome was described in 1958 by E. C. Heyde^1^ as he observed the relation between aortic valve stenosis and gastrointestinal bleeding. In 1992, Warkentin et al.^2^ observed the loss of the largest Willebrand factor multimers, characterizing the Acquired type 2A^3,4^ von Willebrand syndrome. Von Willebrand Factor (vWF) is a multimeric protein, secreted by endothelial cells and platelets.^5^ It promotes adherence of platelets to vascular lesion sites through glycoprotein Ib-vWF interactions. A change in the shape of vWF occurs in AoS, making such protein more susceptible to proteolytic cleavage. As a consequence, vWF is degraded specifically by protease ADAMTS13, hindering vWF-mediated platelet adhesion, thus generating a lack of these multimers and causing bleeding, especially in pre-existing lesions such as gastrointestinal angiodysplasia.^6-8^

Treatment for this syndrome may be obtained with surgical aortic valve replacement, and percutaneous implantation of aortic valve (TAVI), whose effect is still under investigation.^9^

## Case Report

Patient MNS, male, 81 years of age, sought medical treatment for tiredness, black feces, edema of the lower limbs, and worsening of functional class (FC) to NYHA III one month ago.

Patient presents with antecedent systemic arterial hypertension, heart failure, severe AoS, dyslipidemia, diabetes mellitus, chronic renal failure requiring dialysis, two myocardial revascularization surgeries, angioplasty with stent, and anemia.

Physical examination: pale 3+/4+, eupneic, acyanotic. Pulse: 66bpm, BP: 100x60 mmHg, heart rhythm was regular with two clicks with systolic murmur 4+/6+ in the aortic area radiating to the wishbone. Pulmonary auscultation with bibasilar crackles and edema of the lower limbs 2+/4+ with reduced peripheral perfusion.

Labs: Intense anemia with hemoglobin 6.4 g/dL. Initially, the anemia was related to bleeding in the digestive tract due to the black feces - melena. In view of "color anemia" with NYHA III HF, a red blood cell transfusion was requested. 

Transthoracic echocardiography confirmed a double aortic lesion with significant stenosis, with valve area of 0.9 cm^2^ and maximum gradient underestimated of 35 mmHg and mean gradient of 22 mmHg. Ejection fraction of 32%. Left ventricle with 63mm diastolic diameter and 53 mm systolic diameter.

**Figure 1:**
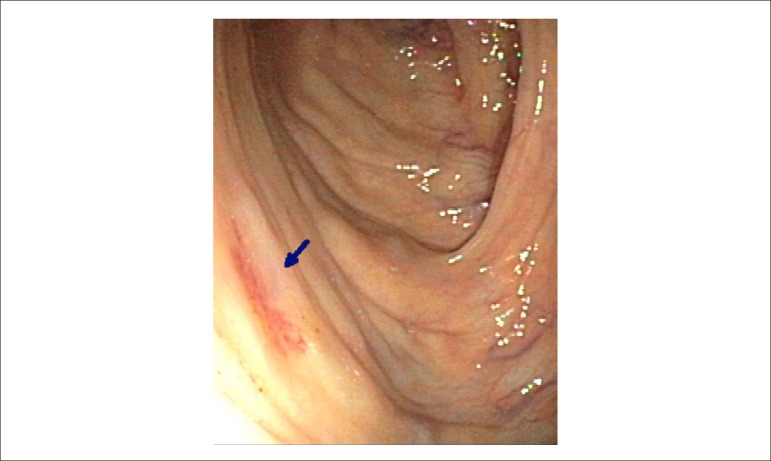
Angiodisplasia de cólon.

Colonoscopy showed the presence of diverticula of sigmoid, descending and transverse colon polyp, and ascending colon angiodysplasia. Thus, the presence of bleeding from angiodysplasia associated to AoS suggested a diagnosis of Heyde's Syndrome (Figure 1).

**Figure 2:**
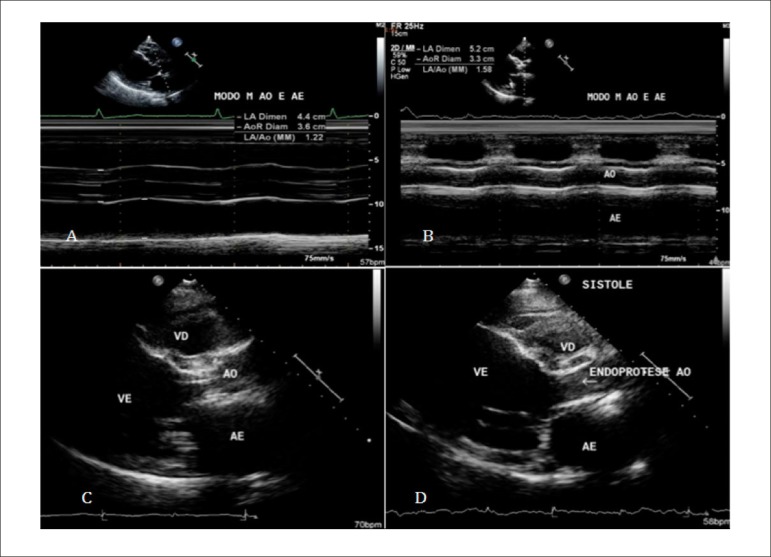
A) ECHO pre TAVI (M mode); B) ECHO post TAVI (M mode); C) ECHO pre TAVI (2D mode); D) ECHO post TAVI (2D mode). AO: aorta; LA: left atrium; RD: right ventricle; LV: left ventricle.

Due to the patient's comorbidities, traditional surgical intervention was discarded due to high risk. A Transcatheter Aortic Valve Implantation (TAVI) was performed, with successful implantation of the transcatheter valve INOVARE^®^ via transapical implantation (Figure 2).

On the fourth day following TAVI, the patient had an episode of enterorrhagia after extreme effort to evacuate his bowels, so blood transfusion was necessary. A colonoscopy and a high digestive endoscopy were then performed with no evidence of active bleeding. Afterwards, the patient was monitored in the clinic, with return visits 3 and 6 months after the surgery with no recurring episodes of bleeding.

## Discussion

Heyde's Syndrome was described in 1958 by Edward C. Heyde as a combination of AoS and bleeding from gastrointestinal angiodysplasia.

The pathophysiology of the condition is explained by the passage of vWF through the stenotic valve, with multimer proteolysis through the enzyme ADAMTS13, a proteinase that acts especially in situations of high shear stress.^3-5,7,8^ VWF is secreted by blood endothelial cells, contributing to the formation of platelet thrombi and acting as a mediator of platelet adhesion in the vascular lesion site.^5^

The relation between AoS and gastrointestinal angiodysplasia has yet to be established. The hypothesis is that AoS is related to a degree of chronic hypoxia, stimulating vasodilation and smooth muscle relaxation, progressing to ecstasia of the vessel wall.^10^ Patients with Heyde's Syndrome treated with bowel resections generally continue to have bleedings in other sites, while the valve approach cures coagulation disorder and anemia.^10^

Elderly patients may present several risks for the surgical replacement of the valve or refuse the procedure. Seniors often have comorbidities that require the use of anti-coagulants or antiplatelet drugs, but those must be avoided, especially in more severe cases. Another option for patients at high surgical risk is the TAVI. 

Recently it was demonstrated that the presence of aortic regurgitation after TAVI can generate multimers proteolysis occurring in some cases the persistence of Heyde's syndrome being associated with a higher mortality at 1 year.^11^

## Conclusion

The elimination of gastrointestinal hemorrhaging risk after calcific aortic valve replacement and valvular prosthesis is well demonstrated in literature.^3-5^

However, there is no evidence that this new approach by TAVI, without removal of the calcium block, can resolve the occurrence of new digestive bleedings. It is necessary, in the long run, to observe and check if the transcatheter valve implantation can correct digestive hemorrhages like the conventional valve replacement.
